# The Prognosis of Males and Females With Moderate or Severe Secondary Mitral Valve Regurgitation and Avenues for Improvement

**DOI:** 10.1111/echo.70370

**Published:** 2025-12-15

**Authors:** Jelle P. Man, Berto J. Bouma, M. A. Molenaar, Steven A. J. Chamuleau, Mark J. Schuuring

**Affiliations:** ^1^ Department of Cardiology Amsterdam UMC Amsterdam the Netherlands; ^2^ Netherlands Heart Institute Utrecht the Netherlands; ^3^ Department of Cardiology Medical Spectrum Twente Enschede the Netherlands; ^4^ Department of Biomedical Signals and Systems University of Twente Enschede the Netherlands

**Keywords:** gender, outcome, valvular heart disease

## Abstract

**Aims:**

To understand prognostic differences between sexes in (subtypes of) secondary mitral valve regurgitation (SMR) and to identify avenues for improvement.

**Method and results:**

In this retrospective study, all consecutive patients diagnosed with moderate or severe SMR by echocardiographic assessment between January 1, 2014, and June 1, 2021 were included. Sex‐specific analyses were performed using Cox proportional hazards analysis, adjusted for significant covariates. A total of 1245 patients with SMR (43% female) were included. Females more often had atrial SMR (233 (29%) females vs. 200 (21%) males, *p* < 0.01), males more often ischemic SMR (100 females (12%) vs. 245 males (25%), *p* < 0.01), and there were no significant differences between sexes in the proportion of non‐ischemic SMR (199 (25%) females vs. 268 (28%) males, *p* = 0.99). The estimated 5‐year survival was 70% (CI = 68%, 73%). Median follow‐up was 4.3 years [2.7–6.2], 236 males and 128 females died during follow‐up. Females had a better survival than males in a multivariable Cox model (HR = 0.67, *p* < 0.01).

**Conclusion:**

Overall survival in patients with SMR was low with an estimated 5‐year survival of 70%. Females had a better survival in patients with SMR than males. The lower survival in males with SMR might be due to a larger proportion of atrial SMR in females, fewer patients with ischemic SMR, and lower ejection fractions in males with non‐ischemic SMR. The current focus on rapid heart failure medication optimization may improve the prognosis of the most vulnerable group; future studies can be directed to see whether this will be the case.

## Graphical abstract

In this Original Research Paper, a sex‐specific analysis, with 43% female patients is performed in a cohort of 1245 patients with secondary mitral valve regurgitation (SMR). In SMR overall survival was low with a 5‐year survival of 70%, the survival was worse in males.

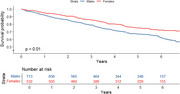

## 1 Introduction

Mitral valve regurgitation (MR) is the most common heart valve disease, and is associated with a substantially increased risk of mortality [1, 2, 3]. Of patients with mitral regurgitation, 65% are classified as secondary mitral valve regurgitation [4], i.e., the regurgitation that occurs due to abnormal geometry of the atrium or ventricle [5]. Contemporary understanding of secondary mitral valve regurgitation (SMR) characterizes the distinct subtypes of ischemic ventricular SMR, non‐ischemic ventricular SMR and atrial SMR. These subtypes have distinct pathophysiological causes, clinical presentation, and therapeutic options [4, 6]. Differences exist between the prevalence of these subtypes of MR between males and females, as males more often have ventricular SMR and females more often have atrial SMR [5, 7, 8]. However, little is known about sex differences in the prognosis of SMR especially between different subtypes, as existing studies are hampered by only selecting patients with heart failure (HF) or by not differentiating by subtype [9, 10].

Understanding the prognostic differences between sexes in SMR is essential to identify areas of improvement and caveats in disease management. Females and males are found to have a distinct reaction to ischemic events and have different cardiac remodeling patterns. Hormonal differences especially linked to estrogen are believed to have an important role in this, due to their ability to induce vasodilatation and reduce left ventricular (LV) remodeling. The differences in cardiac remodeling could also play a role in the prognosis of SMR. The objective of this study was to analyze all‐comer outpatients with SMR, assess prognostic differences between sexes across different pathophysiological subtypes and identify avenues for improvement.

## 2 Methods

All outpatients consecutively diagnosed with moderate to severe SMR by echocardiographic assessment between January 1, 2014 and June 1, 2021 in the outpatient clinic of the Amsterdam University medical center (2 sites) were included. Patients with an assessment of a grade 2 ≥ MR by transthoracic echocardiography (TTE) were followed from their first echocardiogram. Patients with moderate to severe mitral valve stenosis, poor quality TTE assessments, with missing LV or mitral valve functional assessments, or a primary etiology were excluded. This retrospective cohort study was approved by the local institutional review board, who waived the need for written informed consent because of its retrospective and large‐scale design and biases that occur due to the gathering of informed consent.

### 2.1 Data Collection

Baseline and treatment data of patients with MR were collected using chart review of electronic health records (EHR). Data on sex were entered at registration in the hospital's patient portal. TTE assessments were done using Philips or GE systems (Philips Epiq, Philips Affiniti and Philips IE33, Philips Medical Systems, Best, The Netherlands; Vivid, GE Vingmed Ultrasound AS, Horten, Norway).

The initial TTE assessment was performed qualitatively by a clinical technician or cardiology resident in routine clinical practice, and was not performed specifically for this study. Assessments of the heart valves and left ventricular (LV) function were performed according to ESC guidelines. Semi‐quantitative and quantitative measurements of stenosis or regurgitation were obtained to determine the degree of stenosis and/or regurgitation according to the European Society of Cardiology (ESC) guidelines [5]. MR was determined to be moderate or severe. For the functional assessment of the LV the biplane Method of Disks (modified Simpson's rule) was used according to ESC guidelines. LV ejection fraction (LVEF) was classified according to preserved ejection fraction (LVEF ≥ 51% for males and ≥ 53% for females), mildly reduced left ventricular ejection fraction (LVEF < 51% for males and < 53% for females), and reduced left ventricular ejection fraction (LVEF ≤ 40%). Multivalvular disease was defined as regurgitation and/or stenosis in 2 ≤ heart valves [11].

Patients with mitral valve regurgitation and a normal mitral valve structure were considered to have SMR [12]. In cases with multiple identifiable etiologies the clinical status (including patient history and additional imaging by CT or coronary angiography) was assessed by a dedicated imaging cardiologist to determine the most important etiology. Patients with preserved LVEF without regional wall motion abnormalities or leaflet tethering, no or mildly dilated LV cavity, mitral annulus dilatation and an enlarged left atrium were classified as patients with atrial SMR [12]. Patients with ventricular SMR, were further subdivided into patients with an ischemic or non‐ischemic cardiomyopathy. Patients with ischemic cardiomyopathy had prior coronary revascularization with percutaneous coronary intervention or coronary artery bypass grafting or had coronary artery disease diagnosed by their treating physician.

### 2.2 Follow‐Up and Outcome

Mortality rates, the primary outcome of this analysis, were collected using chart review of EHR. The follow‐up started at the date of the first TTE that showed a significant secondary MR. The median and interquartile range of the follow‐up duration will be reported.

### 2.3 Statistical Analysis

Patient characteristics were reported as means and standard deviation (SD) for normally distributed data, and median and interquartile range (IQR) for not normally distributed data. Categorical data were reported using numbers and percentages. For continuous variables Student's *t*‐test or Mann–Whitney *U* tests, if appropriate, was used to test differences in baseline characteristics between groups, and Chi‐square tests were performed for categorical variables. Missing values were multiply imputed using multiple imputation by chained equation (MICE) and predictive mean matching. The number of performed imputations was 12.

Survival analyses were performed using Cox proportional hazards models, and visualized using Kaplan–Meier curves. The degree to which survival differed between MR subtypes was analyzed separately. The subtypes are included in the multivariate model without interaction terms, as such a model would be hard to interpret clinically. A covariate adjusted analysis was done to account for differences in baseline characteristics among males and females. The survival analysis was performed on the imputed dataset and a sensitivity analysis was conducted by performing a complete case analysis. Covariates were selected based on hypothesized importance. Selected covariates with a two‐sided *p* value < 0.05 in the univariate Cox proportional hazards analysis were considered statistically significant and were included in the multivariable Cox proportional hazards model. The hazard ratios and confidence intervals are reported. The concordance index is also calculated and reported to evaluate the predictive capability of the model. To evaluate the assumption of proportional hazards, Kaplan–Meier curves were inspected, and Schoenfeld residuals were calculated. The number of events per covariate was reported to ensure that the model is not overfitted.

Statistical analyses were performed in RStudio V2024.04.2 (RStudio Team, Boston, Massachusetts) using R‐version 6.2.5.1 (R Core Team, Vienna, Austria). A two‐sided *p* < 0.05 was considered statistically significant.

## 3 Results

**FIGURE 1:**
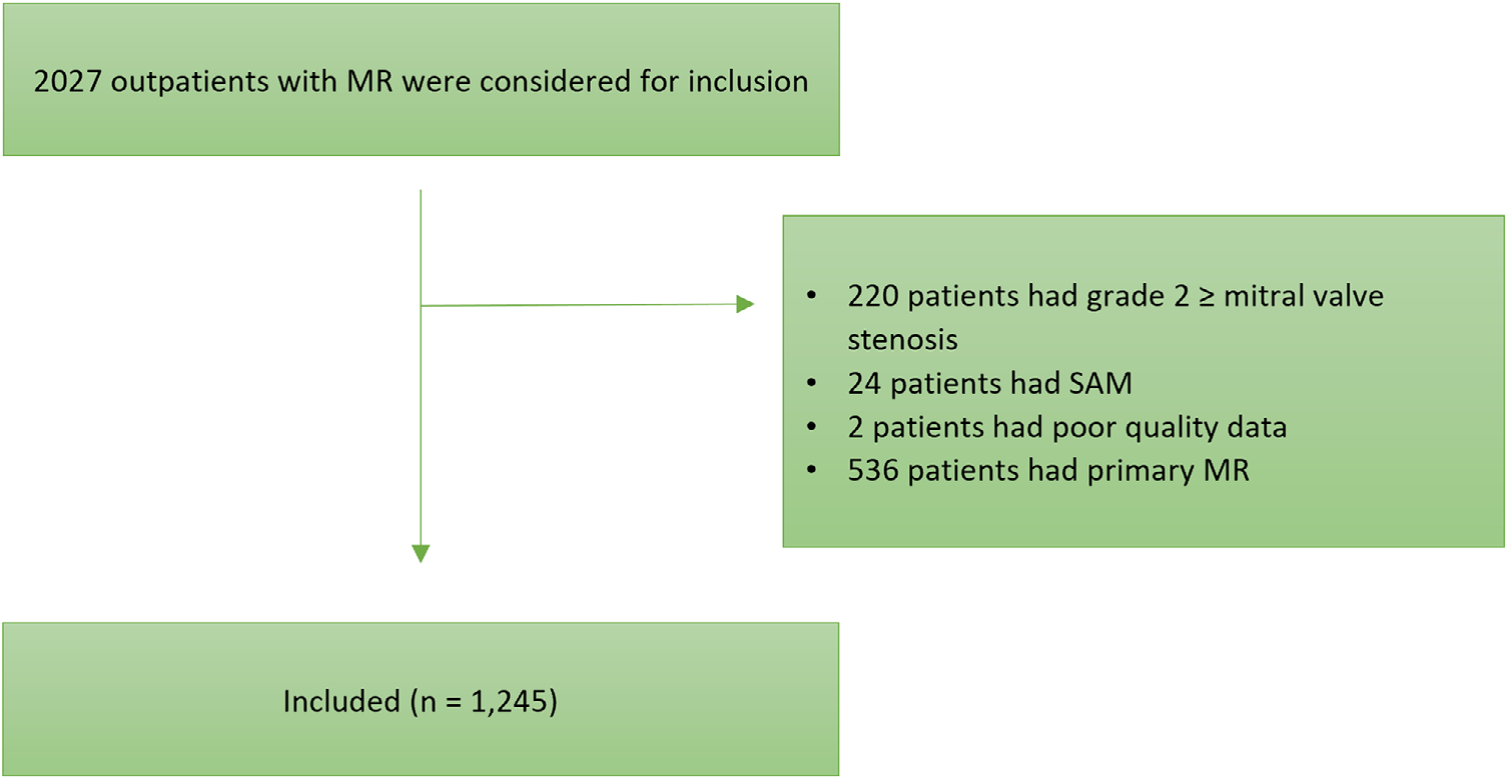
Patient flow diagram for inclusion in this cohort.

A total of 1245 outpatients (43% female) with SMR were included. Females were older (75 years [65, 83] vs. 73 years [63, 80], *p* < 0.01) Figure 1. Females more frequently presented with atrial SMR (233 [29%] females vs. 200 [21%] males, *p *< 0.01), males more frequently presented with ischemic SMR (100 females [12%] vs. 245 males [25%], *p* < 0.01), and there were no significant differences between sexes in the proportion of non‐ischemic SMR (199 [25%] females vs. 268 [28%] males, *p* = 0.99). The baseline characteristics are shown in Table 1. In non‐ischemic SMR, females had lower NT‐proBNP values (1623 [835, 4014] in females vs. 2767 [1262, 5996] in males, *p* = 0.01), and less often a reduced left ventricular ejection fraction (209 [32%] vs. 424 [51%], *p* < 0.01). In atrial SMR, concomitant moderate to severe tricuspid regurgitation was more common among females (44 [20.0%] vs. 18 males [9.2%], *p* < 0.01). In ischemic SMR, atrial flutter or fibrillation was less common among females (24 [25%] vs. 113 [46%], *p* < 0.01). The baseline characteristics of the subtypes of SMR are displayed in Table 2. Data imputation was performed for the following variables: NT‐proBNP 30% missing, eGFR 24% missing, and BMI 20% missing (Supporting Information).

**TABLE 1:** Baseline characteristics stratified by type of MR and sex.

	SMR		
	Male	Female	*p*
*n*	713	532	
Age (median [IQR])	73 [63, 80]	75 [65, 83]	<0.01
BMI (median [IQR])	26 [24, 29]	26 [23, 30]	0.70
Mitral valve regurgitation grade			0.11
Moderate	578 (94)	487 (81)	
Severe	44 (6)	46 (9)	
Hypertension (%)	280 (39)	246 (46)	<0.01
Diabetes (%)	173 (24)	106 (20)	0.091
Reduced LVEF	390 (54)	189 (36)	<0.01
Use of loop diuretics (%)	399 (56)	310 (58)	0.43
HF medication use among reduced LVEF			
RASi (%)	257 (68)	140 (75)	0.22
β‐blocker (%)	289 (77)	142 (76)	0.93
MRA (%)	177 (47)	81 (44)	0.66
Atrial fibrillation or flutter (%)	308 (43)	196 (37)	0.026
eGFR (median [IQR])	60 [44, 71]	60.00 [44, 70]	0.78
NTproBNP (median [IQR])	2,564 [928, 6,238]	1,786 [752, 4,065]	< 0.01
More than 1 affected valve (%)	175 (25)	161 (30)	0.029
AS(%)	64 (9.0)	58 (10)	0.30
AR (%)	55 (7.7)	38 (7.1)	0.79
TR (%)	82 (11)	93 (18)	< 0.01
Type of SMR			
Atrial SMR (%)	200 (28)	233 (44)	< 0.01
Non‐ischemic SMR (%)	268 (38)	199 (37)	0.99
Ischemic SMR(%)	245 (34)	100 (19)	< 0.01

Abbreviations: AS, aortic valve stenosis; AR, aortic valve regurgitation; BMI, body mass index; IQR, interquartile rang; LV, left ventricle; MR, mitral valve regurgitation; PMR, primary MR; SMR, secondary MR; TR, tricuspid valve regurgitation.

**TABLE 2:** Baseline characteristics of SMR subdivided in its contemporary subtypes.

	Non‐ischemic SMR			Ischemic SMR			Atrial SMR		
	Male	Female	*p*	Male	Female	*p*	Male	Female	*p*
*n*	268	199		245	100		200	233	
Age (median [IQR])	70 [60, 78]	73 [62, 82]	0.02	74 [67, 80]	75 [68, 83]	0.11	75 [67, 82]	78 [69, 84]	0.057
BMI (median [IQR])	26 [24, 29]	25.50 [22, 31]	0.69	26 [23, 29]	25 [23, 29]	0.74	26 [24, 29]	26 [23, 29]	0.99
Mitral valve regurgitation grade (%)			0.21			0.37			0.03
Moderate	244 (91)	174 (87)		227 (92)	88 (88)		198 (94)	224 (86)	
Severe	24 (9)	25 (13)		18 (8)	12 (12)		2 (1)	9 (4)	
Hypertension (%)	99 (37)	88 (44)	0.23	80 (34)	34 (34)	0.90	92 (47)	113 (51)	0.31
Diabetes (%)	54 (20)	45 (22)	0.56	75 (31)	26 (26)	0.58	40 (21)	33 (15)	0.18
Heart failure (%)	236 (88)	169 (84)	0.40	234 (95)	95 (95)	1.00	128 (64)	146 (62)	0.85
Reduced LVEF (%)	191 (71)	113 (57)	< 0.01	196 (80)	76 (76)	0.66			
Atrial fibrillation or flutter (%)	107 (40)	69 (35)	0.18	113 (46)	24 (25)	< 0.01	84 (43)	95 (43)	1, 000
eGFR (median [IQR])	60 [44, 70]	59 [40, 73]	0.35	56 [40, 68]	56 [42, 62]	0.71	61 [48, 78]	60 [48, 74]	0.4
NTproBNP (median [IQR])	2,767 [1,262, 5,996]	1,623 [835, 4,014]	0.01	3,375 [1,333, 7,633]	3,149 [1,327, 7,384]	0.99	976 [407, 3,881]	1,289 [489, 2,339]	0.95
Use of loop diuretics (%)	164 (61)	132 (66)	0.30	159 (64)	68 (68)	0.67	76 (38)	110 (47)	0.061
Medication use for heart failure									
RASi (%)	166 (61)	135 (67)	0.22	169 (69)	67 (67)	0.82	98 (49)	112 (48)	0.92
β‐blocker (%)	189 (70)	139 (69)	0.96	195 (80)	74 (74)	0.32	116 (58)	147 (63)	0.326
MRA (%)	92 (34)	65 (32)	0.78	110 (45)	39 (39)	0.38	23 (12)	32 (14)	0.581
More than 1 affected valve (%)	68 (25)	58 (29)	0.46	54 (23)	18 (19)	0.46	50 (26)	84 (36)	0.03
AS (%)	17 (6.3)	20 (10.1)	0.20	20 (8.2)	4 (4.0)	0.25	27 (14)	34 (15)	0.85
AR (%)	27 (10.1)	14 (7.0)	0.33	14 (5.7)	4 (4.0)	0.70	14 (7.0)	20 (8.6)	0.66
TR (%)	35 (13.1)	33 (16.6)	0.35	28 (11)	13 (13)	0.82	19 (9.5)	47 (20)	< 0,01

Abbreviation: AS, aortic valve stenosis; AR, aortic valve regurgitation; BMI, body mass index; eGFR, estimated glomerular filtration rate; IQR, interquartile range; LVEF, left ventricular ejection fraction; SMR, secondary mitral valve regurgitation; TR, tricuspid valve regurgitation.

**TABLE 3:** Univariable and multivariable Cox regression of covariates.

	Univariate analysis		Multivariate analysis	
	Hazard ratio (95% Confidence interval)	*p* value	Hazard ratio (95% Confidence interval)	*p* value
Sex (male)	0.65 (0.52, 0.80)	< 0.01	0.69 (0.52, 0.91)	< 0.01
Age	1.02 (1.01, 1.03)	< 0.01	0.998 (0.996, 1.004)	0.46
BMI	1.00 (0.98, 1.03)	0.79		
Severity of MR regurgitation	1.31 (1.12, 1.53)	< 0.01	1.08 (0.88, 1.31)	0.45
Hypertension	0.78 (0.63, 0.96)	0.023		
Diabetes	1.62 (1.29, 2.03)	< 0.01	1.53 (1.16, 2.03)	< 0.01
LVEF	1.55 (1.37, 1.76)	< 0.01	1.19 (0.94, 1.51)	0.15
NT‐proBNP	1.00 (1.00, 1.00)	< 0.01	1.000026 (1.000016, 1.0000361)	< 0.01
reduced kidney function	2.50 (1.95, 3.20)	< 0.01	1.76 (1.33, 2.33)	< 0.01
Atrial fibrillation	1.41 (1.15, 1.73)	< 0.01	1.40 (1.08, 1.81)	0.01
Atrial SMR	0.57 (0.45, 0.72)	< 0.01	1.03 (0.65, 1.63)	0.89
Ischemic SMR	1.55 (1.25, 1.92)	< 0.01	1.08 (0.81, 1.44)	0.60
Non‐ischemic SMR	1.12 (0.91, 1.38)	0.30		
Multivalve disease	1.70 (1.38, 2.11)	< 0.01	1.48 1.12 1.95	< 0.01
Transcatheter intervention	1.69 (0.90, 3.17)	0.10		
Surgical intervention	0.60 (0.08, 4.29)	0.61		

Abbreviations: BMI, body mass index; LVEF, left ventricular ejection fraction; MR, mitral valve regurgitation; SMR, secondary MR.

### 3.1 Therapy for Mitral Valve Regurgitation

Among patients with a reduced left ventricular ejection fraction, 77% received a β‐blocker, 68% a Renin angiotensin system inhibitor (RASi), and 46% received a mineralocorticoid receptor antagonist (MRA); there were no differences between sexes (Table 1). In this cohort 11 males (1.5%) and 8 females (1.5%) received transcatheter mitral valve repair; there were no differences between sexes (*p* = 1.00). Surgery was rarely performed; there were no differences between sexes (1 (0.1%) male and 4 (0.8%) females, *p* = 0.21). We found no differences in the interval from diagnosis to referral (median [IQR] = 292 [65, 1706] vs. 701 [209, 1395], *p* = 0.42).

### 3.2 Survival Analysis

**FIGURE 2:**
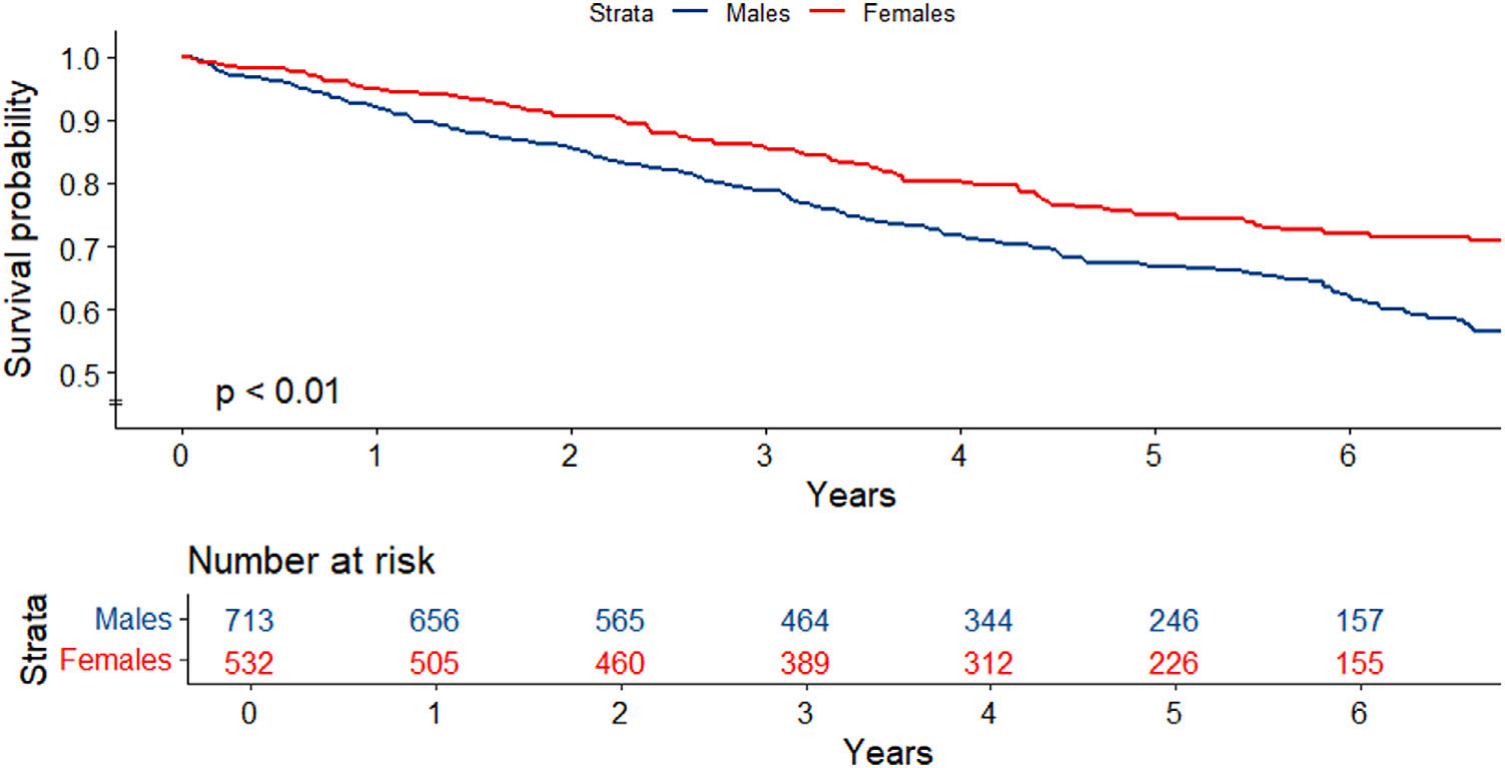
This figure shows the survival of males and females with SMR. Females with SMR had a better survival than males (*p* < 0.01).

**FIGURE 3:**
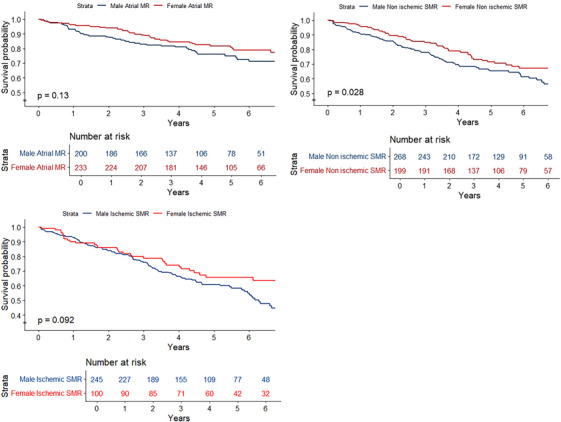
SMR subdivided in its contemporary subtypes (non‐ischemic ventricular MR, ischemic ventricular MR and atrial MR) and stratified by sex. Males with non‐ischemic SMR had a worse prognosis compared to females (*p* = 0.028), while no differences were found in the survival in atrial SMR and ischemic SMR.

The estimated 5‐year survival was 70% CI = (68%, 73%). The estimated survival for females was 75% (71%, 79%), and 67% (63%, 71%) for males. Females had in a multivariable cox model a better survival than males (HR = 0.69, *p* < 0.01, Figure 2, Table 3. This result remained consistent in the complete‐case sensitivity analysis. The assumptions of the cox proportional hazards model were met. The concordance index was 0.68. The number of events per covariate was 30. Median follow‐up was 4.3 years [2.7–6.2], 236 males and 128 females died during follow‐up. In subtypes of SMR, cox proportional hazards analyses showed that females had a better survival in non‐ischemic SMR (HR = 0.69, *p* = 0.029; Figure 3. There were no significant differences between the survival of male and female patients with atrial SMR (*p* = 0.13) or ischemic SMR (*p* = 0.092).

## 4 Discussion

In this retrospective cohort study, we analyzed all‐comer outpatients with SMR and identified differences regarding prognosis among different pathophysiological (sub)types. Overall survival in patients with SMR was low with an estimated 5‐year survival of 70%. Females with SMR had in a multivariable cox model a better survival than males (HR = 0.69, *p* < 0.01). The hazard ratio for males compared with females attenuated from 0.65 in the unadjusted model to 0.69 after adjusting for confounding variables. This attenuation suggests that the crude association between sex and survival was only to limited extent confounded by these variables.

The concordance index of our model was 0.68. This indicates that the model had a moderate capability to distinguish patients with a poor outcome vs. patients with a good outcome. We believe this is an acceptable concordance index for our purposes, since real world baseline clinical data can only to a limited extent predict survival. More genetic, longitudinal and historical data along with non‐linear models can be used to provide a better prediction of survival in patients with SMR, but this is outside the scope of this study. The number of interventions in our study is low, but this is understandable as the 2021 ESC/EACTS guidelines only recommend surgery if patients are suitable for surgery, have complaints while fully up‐titrated in their HF medication, have a longer than 1‐year life expectancy, and have no other cardiac disease requiring treatment. This, of course, rarely occurs in patients who are receiving follow‐up in an outpatient clinic.

Pathophysiological differences between sexes exist, as registries have indicated sex differences in the prevalence of subtypes of SMR. Registries in Australia, Japan, and Olmsted County, USA, have indicated that the prevalence of subgroups of MR differs between sexes. In these registries men more often have ventricular SMR and females more often have atrial SMR [5, 7, 8]. We find similar proportions of ventricular SMR (66%) and atrial SMR (34%) in this (to our knowledge first) European cohort reporting on these subtypes. These registries are focused on the contemporary subtypes of MR (and their outcomes) and do not focus on prognostic differences between sexes or factors explaining pathophysiological differences between sexes.

There have been a limited number of studies focused on prognostic differences between sexes. Benfari et al. reported on the differences in survival between male and female patients, but did not differentiate their patients into contemporary subgroups or explore potential causes related to these differences, as their study had a different aim [10]. In a Dutch cohort of 698 patients with HF and moderate or severe SMR prognostic differences between sexes were assessed [9]. Females were found to have an overall better survival compared to males. However, since this study only included patients with HF (60% had NYHA class III), the role of patients with atrial SMR or patients without HF is not taken into account. As the present study included all patients with SMR, this cohort provides complementary information on all‐comers prognostic differences in different pathophysiological (sub)types of SMR found in the outpatient clinic. Females with SMR had in our multivariable cox model a better survival than males (HR = 0.69, *p* < 0.01).

From our cohort, it is apparent that patients with ischemic SMR have a low survival and overall poorer LV functional assessments than other types of SMR (lower NT‐proBNP values and more patients with a reduced LVEF, Table 1). The high proportion of males in this ischemic SMR group is likely to be an important factor in the overall lower survival among males with SMR. We also find a lower survival of males than females in patients with non‐ischemic SMR (*p* = 0.029). In these patients with non‐ischemic SMR, males also had significantly worse LV functional assessments than females (Table 2). Among patients with SMR and a reduced left ventricular ejection fraction the use of GDMT was low; 76% received a β‐blocker, 71% a RASi, and 46% received a MRA (Table 1).

### 4.1 Future Directions and Implications

With the current focus on rapid optimization, future studies can be directed to see if a focus on rapid optimization can improve the poor prognosis of the vulnerable groups of patients with SMR. Optimization strategies using digital solutions may be effective to increase these prescription rates [13, 14, 15, 16]. Also, there is a clear distinction in the pathophysiological mechanisms that underlie the survival differences. The found mortality rate may justify an enhanced focus on the vulnerable patients, focusing on rapid referrals to HF clinics for GDMT optimization or on rapid up‐titration by the imaging cardiologist, when a patient with SMR is encountered. Additionally, the distinct profiles found in males and females emphasize the need to develop distinct therapies suitable for each respective phenotype.

### 4.2 Limitations

This cohort study was limited by its retrospective design. The cause of mortality was not for all patients available and was therefore not further differentiated. This could dilute disease‐specific associations of mortality. As we analyzed outpatients, deaths occurred outside of the hospital and reasons for deaths are often not investigated. Females were older in our cohort, and this could be a source of unexplained mortality. However, despite the extra deaths that may result from a higher age, females still had a better survival. Also, we accounted for several relevant confounding conditions or diseases that may have an impact on mortality. This increases the likelihood that the survival rates were associated with the disease. This large retrospective analysis in patients with SMR was, to our knowledge, the first analyze prognostic differences in contemporary subtypes of SMR between sexes in all‐comers in outpatient clinics.

## 5 Conclusion

Overall survival in patients with SMR was low with an estimated 5‐year survival of 70%. Females had a better survival in patients with SMR than males. The lower survival in males with SMR might be due to a larger proportion of atrial SMR in females, less patients with ischemic SMR, and lower ejection fractions in males with non‐ischemic SMR. The current focus on rapid HF medication optimization might improve the prognosis of the most vulnerable group; future studies can be directed to see if this will be the case.

## Conflicts of Interest

M. J. S. acknowledges to be a member of a Dutch CardioVascular Alliance consortium (ADMINISTER II), which is supported by public/not‐for‐profit organizations (i.a. Stichting Hartcentrum Twente) and partners with independent contributions to the research institute (AstraZeneca & Boehringer Ingelheim). M. J. S. also received grant funding from the Pioneers in Healthcare scheme (University of Twente) and TKI‐PPP (Health Holland).

## Supporting information




**Supplementary Materials**: Comparison of distributions of imputed vs complete datasets.

## References

[1] J. L. D'Arcy , S. Coffey , M. A. Loudon , et al., “Large‐Scale Community Echocardiographic Screening Reveals a Major Burden of Undiagnosed Valvular Heart Disease in Older People: The OxVALVE Population Cohort Study,” European Heart Journal 37 (2016): 3515–3522. 27354049 10.1093/eurheartj/ehw229 PMC5216199

[2] V. T. Nkomo , J. M. Gardin , T. N. Skelton , J. S. Gottdiener , C. G. Scott , and M. Enriquez‐Sarano , “Burden of Valvular Heart Diseases: A Population‐Based Study,” Lancet 368 (2006): 1005–1011. 16980116 10.1016/S0140-6736(06)69208-8

[3] V. Dziadzko , M. Clavel , M. Dziadzko , et al., “Outcome and Undertreatment of Mitral Regurgitation: A Community Cohort Study,” Lancet 391 (2018): 960–969. 29536860 10.1016/S0140-6736(18)30473-2 PMC5907494

[4] A. Vahanian , F. Beyersdorf , F. Praz , et al., “2021 ESC/EACTS Guidelines for the Management of Valvular Heart Disease Developed by the Task Force for the Management of Valvular Heart Disease of the European Society of Cardiology (ESC) and the European Association for Cardio‐Thoracic Surgery (EACTS),” European Heart Journal 43, no. 7 (2021): 561–632, 10.1093/EURHEARTJ/EHAB395.

[5] V. Dziadzko , M. Dziadzko , J. R. Medina‐Inojosa , et al., “Causes and Mechanisms of Isolated Mitral Regurgitation in the Community: Clinical Context and Outcome,” European Heart Journal 40 (2019): 2194–2202. 31121021 10.1093/eurheartj/ehz314

[6] A. El Sabbagh , Y. N. V. Reddy , and R. A. Nishimura , “Mitral Valve Regurgitation in the Contemporary Era: Insights Into Diagnosis, Management, and Future Directions,” Journal of the American College of Cardiology Cardiovasc Imaging 11 (2018): 628–643. 10.1016/j.jcmg.2018.01.009 29622181

[7] K. Kim , T. Kitai , S. Kaji , et al., “Outcomes and Predictors of Cardiac Events in Medically Treated Patients With Atrial Functional Mitral Regurgitation,” International Journal of Cardiology 316 (2020): 195–202. 32610155 10.1016/j.ijcard.2020.06.042

[8] A. Moonen , M. K. C. Ng , D. Playford , et al., “Atrial Functional Mitral Regurgitation: Prevalence, Characteristics and Outcomes From the National Echo Database of Australia,” Open Heart 10 (2023): e002180. 36792312 10.1136/openhrt-2022-002180 PMC9933756

[9] F. Namazi , P. van der Bijl , N. M. Vo , et al., “Sex Differences in Prognosis of Significant Secondary Mitral Regurgitation,” European Society of Cardiology Heart Failure 8 (2021): 3539–3546. 10.1002/ehf2.13503 PMC8497350 34363328

[10] J. Avierinos , J. Inamo , F. Grigioni , B. Gersh , C. Shub , and M. Enriquez‐Sarano , “Sex Differences in Morphology and Outcomes of Mitral Valve Prolapse,” Annals of Internal Medicine 149 (2008): 787–794. 19047025 10.7326/0003-4819-149-11-200812020-00003 PMC2897166

[11] P. Unger , M. Clavel , B. R. Lindman , P. Mathieu , and P. Pibarot , “Pathophysiology and Management of Multivalvular Disease,” Nature Reviews Cardiology 13 (2016): 429–440. 27121305 10.1038/nrcardio.2016.57 PMC5129845

[12] F. Praz , M. A. Borger , J. Lanz , et al., “ESC/EACTS Guidelines for the Management of Valvular Heart Disease: Developed by the Task Force for the Management of Valvular Heart Disease of the European Society of Cardiology (ESC) and the European Association for Cardio‐Thoracic Surgery (EACTS),” European Heart Journal 46 (2025): 4635–4736, 10.1093/eurheartj/ehaf194. 40878295

[13] D. H. Brahmbhatt , H. J. Ross , M. O'Sullivan , et al., “The Effect of Using a Remote Patient Management Platform in Optimizing Guideline‐Directed Medical Therapy in Heart Failure Patients: A Randomized Controlled Trial,” Journal of the American College of Cardiology Heart Failure 12 (2024): 678–690. 10.1016/j.jchf.2024.02.008 38569821

[14] J. P. Man , J. Klopotowska , F. W. Asselbergs , M. L. Handoko , S. A. J. Chamuleau , and M. J. Schuuring , “Digital Solutions to Optimize Guideline‐Directed Medical Therapy Prescriptions in Heart Failure Patients: Current Applications and Future Directions,” Current Heart Failure Reports 21 (2024): 147–161. 38363516 10.1007/s11897-024-00649-x PMC10924030

[15] J. P. Man , M. A. C. Koole , P. G. Meregalli , et al., “Digital Consults in Heart Failure Care: A Randomized Controlled Trial,” Nature Medicine (2024), 10.1038/s41591-024-03238-6. PMC11485254 39217271

[16] M. J. Schuuring , J. P. Man , and S. A. J. Chamuleau , “Inclusive Health Tracking,” Journal of the American College of Cardiology Advances 2 (2023): 100545. 38939485 10.1016/j.jacadv.2023.100545 PMC11198698

