# Supplementary figures and images for: The Prognosis of Males and Females With Moderate or Severe Secondary Mitral Valve Regurgitation and Avenues for Improvement
Source: Echocardiography. 2025 Dec 15;42(12):e70370. doi: 10.1111/echo.70370 (PMC12704036; doi:10.1111/echo.70370)

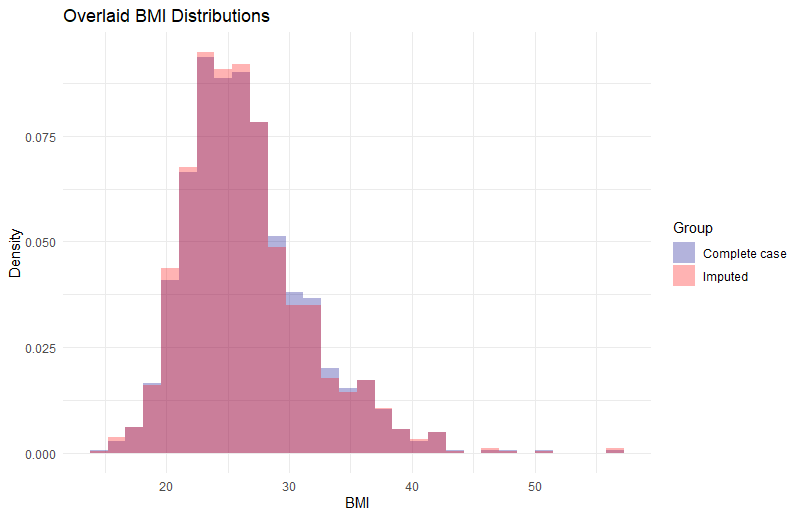
Supplementary materials
**comparison of distributions of imputed vs complete datasets**


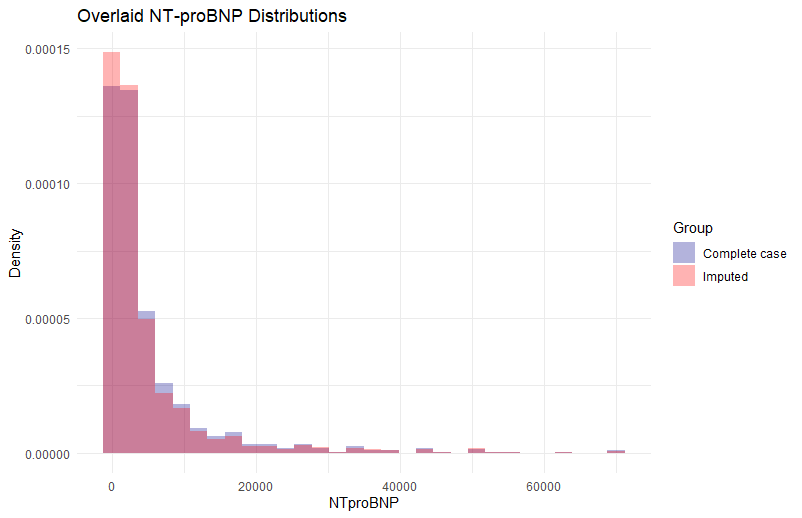


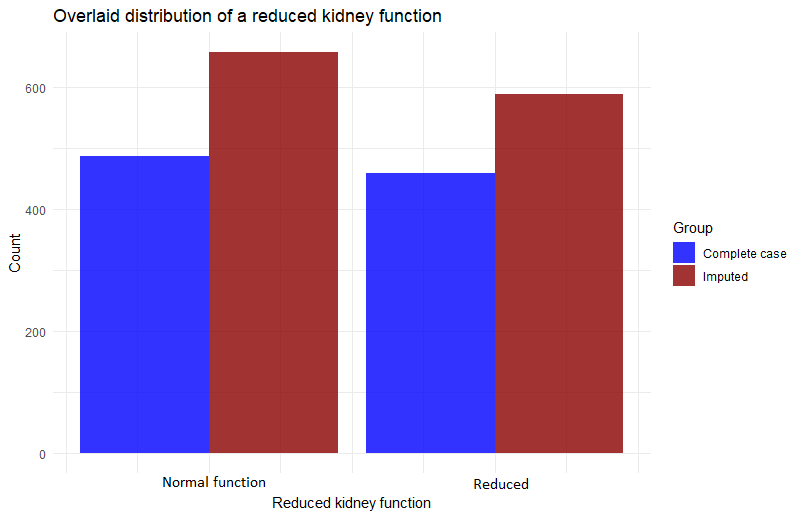

Supplement: Supplementary file 1 — Supplementary Materials: Comparison of distributions of imputed vs complete datasets. [file ECHO-42-e70370-s001.docx]
